# Supplementary material for: MITF Contributes to the Body Color Differentiation of Sea Cucumbers Apostichopus japonicus through Expression Differences and Regulation of Downstream Genes
Source: Biology (Basel). 2022 Dec 20;12(1):1. doi: 10.3390/biology12010001 (PMC9854957; doi:10.3390/biology12010001)
Supplement: Supplementary file 1 [file biology-12-00001-s001.zip › biology-2049586-supplementary/Table S7.pdf]

Table S7 HOMER enrichment results for known motifs in purple *A. japonicus*, compared with green *A. japonicus*.

**Rank**	**Motif**	**Log (*p* value)**	**Name**
1	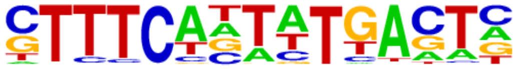	-1.173e+01	IRF:BATF(IRF:bZIP)/pDC-Irf8-ChIP-Seq(GSE66899)/Homer
2	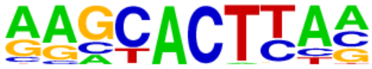	-9.468e+00	Nkx3.1(Homeobox)/LNCaP-Nkx3.1-ChIP-Seq(GSE28264)/Homer
3	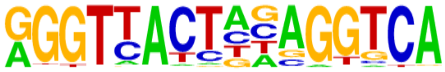	-9.079e+00	LXRE(NR),DR4/RAW-LXRb.biotin-ChIP-Seq(GSE21512)/Homer
4	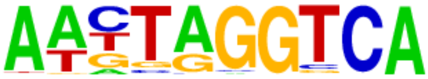	-7.664e+00	RORgt(NR)/EL4-RORgt.Flag-ChIP-Seq(GSE56019)/Homer
5	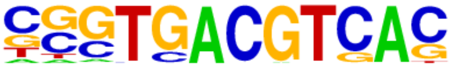	-7.281e+00	CRE(bZIP)/Promoter/Homer
6	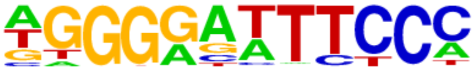	-6.645e+00	NFkB-p65(RHD)/GM12787-p65-ChIP-Seq(GSE19485)/Homer
7	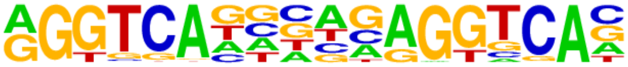	-6.378e+00	RAR:RXR(NR),DR5/ES-RAR-ChIP-Seq(GSE56893)/Homer
8	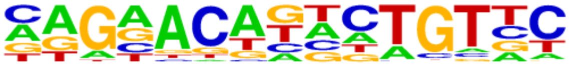	-6.274e+00	PR(NR)/T47D-PR-ChIP-Seq(GSE31130)/Homer
9	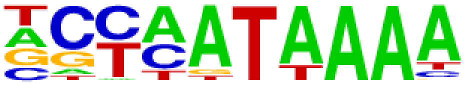	-5.540e+00	HOXD13(Homeobox)/Chicken-Hoxd13-ChIP-Seq(GSE38910)/Homer
10	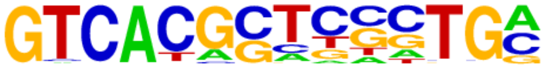	-5.127e+00	PAX5(Paired,Homeobox),condensed/GM12878-PAX5-ChIP-Seq(GSE32465)/Homer
11	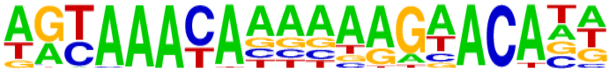	-4.705e+00	FOXA1:AR(Forkhead,NR)/LNCAP-AR-ChIP-Seq(GSE27824)/Homer
