# Supplementary material for: MITF Contributes to the Body Color Differentiation of Sea Cucumbers Apostichopus japonicus through Expression Differences and Regulation of Downstream Genes
Source: Biology (Basel). 2022 Dec 20;12(1):1. doi: 10.3390/biology12010001 (PMC9854957; doi:10.3390/biology12010001)
Supplement: Supplementary file 1 [file biology-12-00001-s001.zip › biology-2049586-supplementary/Table S8.pdf]

Table S8 HOMER enrichment results for known motifs in white *A. japonicus*, compared with green *A. japonicus*.

**Rank**	**Motif**	**Log (*p* value)**	**Name**
1	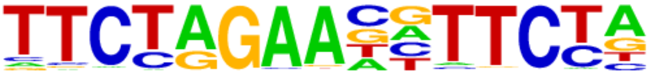	-1.347e+01	HRE(HSF)/Striatum-HSF1-ChIP-Seq(GSE38000)/Homer
2	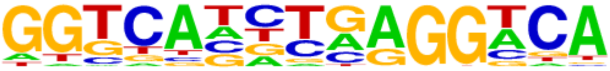	-1.092e+01	THRa(NR)/C17.2-THRa-ChIP-Seq(GSE38347)/Homer
3	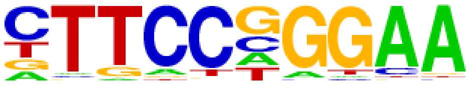	-1.024e+01	Stat3(Stat)/mES-Stat3-ChIP-Seq(GSE11431)/Homer
4	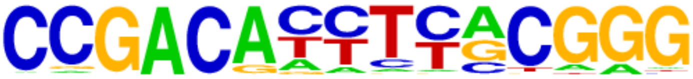	-9.730e+00	GEI-11(Myb?)/cElegans-L4-GEI11-ChIP-Seq(modEncode)/Homer
5	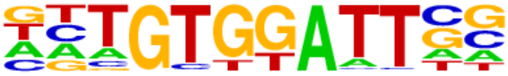	-8.971e+00	Foxh1(Forkhead)/hESC-FOXH1-ChIP-Seq(GSE29422)/Homer
6	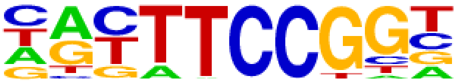	-8.804e+00	Elk4(ETS)/Hela-Elk4-ChIP-Seq(GSE31477)/Homer
7	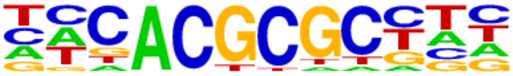	-8.179e+00	FHY3(FAR1)/Arabidopsis-FHY3-ChIP-Seq(GSE30711)/Homer
8	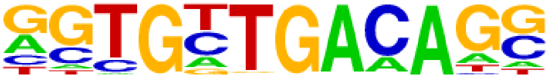	-7.943e+00	Tbx20(T-box)/Heart-Tbx20-ChIP-Seq(GSE29636)/Homer
9	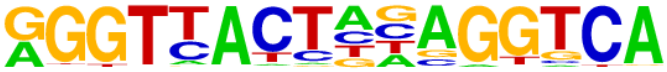	-7.697e+00	LXRE(NR),DR4/RAW-LXRb.biotin-ChIP-Seq(GSE21512)/Homer
10	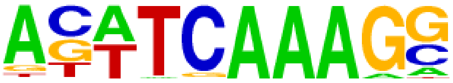	-7.189e+00	Tcf3(HMG)/mES-Tcf3-ChIP-Seq(GSE11724)/Homer
11	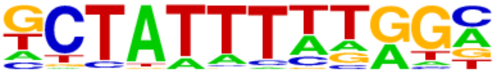	-6.747e+00	Mef2b(MADS)/HEK293-Mef2b.V5-ChIP-Seq(GSE67450)/Homer
12	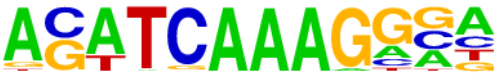	-6.300e+00	Tcf4(HMG)/Hct116-Tcf4-ChIP-Seq(SRA012054)/Homer
13	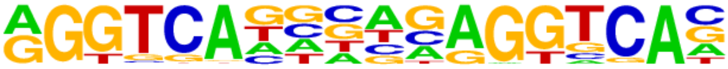	-6.116e+00	RAR:RXR(NR),DR5/ES-RAR-ChIP-Seq(GSE56893)/Homer

14	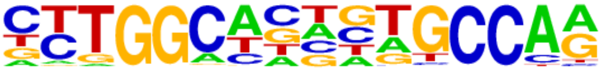	-5.853e+00	NF1(CTF)/LNCAP-NF1-ChIP-Seq(Unpublished)/Homer
15	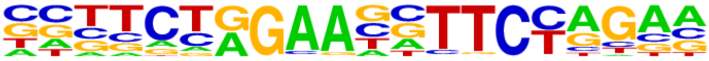	-5.752e+00	HRE(HSF)/HepG2-HSF1-ChIP-Seq(GSE31477)/Homer
16	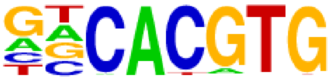	-5.073e+00	BMAL1(bHLH)/Liver-Bmal1-ChIP-Seq(GSE39860)/Homer
17	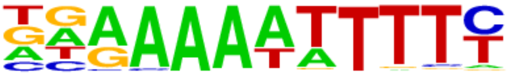	-5.055e+00	SFP1/SacCer-Promoters/Homer
18	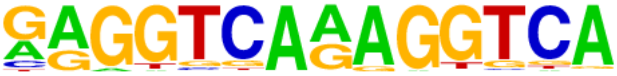	-4.988e+00	TR4(NR),DR1/Hela-TR4-ChIP-Seq(GSE24685)/Homer
19	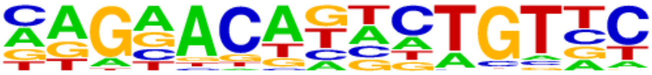	-4.933e+00	PR(NR)/T47D-PR-ChIP-Seq(GSE31130)/Homer
20	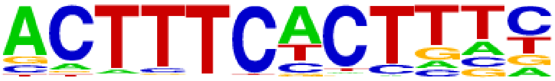	-4.925e+00	PRDM1(Zf)/Hela-PRDM1-ChIP-Seq(GSE31477)/Homer
21	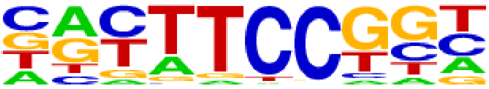	-4.878e+00	Fli1(ETS)/CD8-FLI-ChIP-Seq(GSE20898)/Homer
22	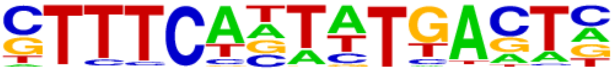	-4.624e+00	IRF:BATF(IRF:bZIP)/pDC-Irf8-ChIP-Seq(GSE66899)/Homer
